# Association between obesity and Barrett’s esophagus in a Japanese population: a hospital-based, cross-sectional study

**DOI:** 10.1186/1471-230X-13-143

**Published:** 2013-09-26

**Authors:** Jiro Watari, Kazutoshi Hori, Fumihiko Toyoshima, Noriko Kamiya, Takahisa Yamasaki, Takuya Okugawa, Haruki Asano, Zhao Liang Li, Takashi Kondo, Hisatomo Ikehara, Jun Sakurai, Toshihiko Tomita, Tadayuki Oshima, Hirokazu Fukui, Hiroto Miwa

**Affiliations:** 1 Division of Upper Gastroenterology, Department of Internal Medicine, Hyogo College of Medicine, 1-1, Mukogawa-cho, Nishinomiya 663-8501, Japan

**Keywords:** Barrett’s esophagus, Body mass index, Waist circumference, Visceral obesity, Reflux esophagitis

## Abstract

**Background:**

The association between obesity and Barrett’s esophagus (BE) in the Japanese population remains unclear. The prevalence of BE and its associated risk factors was examined.

**Methods:**

A cross-sectional study of 1581 consecutive individuals who underwent upper gastrointestinal endoscopy was conducted. The prevalence of endoscopically suspected BE (ESBE) was evaluated. Obesity was evaluated by body mass index (BMI, ≥ 25 kg/m^2^) and waist circumference (WC) (males, ≥ 85 cm; females, ≥ 90 cm). Because endoscopic diagnosis of ultra-short ESBE (<1 cm in extent) is difficult and highly unreliable, this type of ESBE was excluded from the study.

**Results:**

In proton pump inhibitor (PPI) non-users, the prevalence of ESBE ≥ 1 cm was 5.6%. In univariate analysis, male sex and reflux esophagitis (RE) were significantly associated with BE, but BMI, WC, and reflux symptoms were not. In multivariate logistic regression analysis, only RE (odds ratio [OR] = 3.48, 95% confidence interval [CI] 1.89-6.41, p < 0.0001) was an independent risk factor for BE; obesity and the other factors were not. In contrast, RE (OR 5.67, p = 0.0004) and large WC (OR 5.09, p = 0.0005) were significant risk factors for ESBE ≥ 1 cm in PPI users. Only male sex, but not obesity or the other risk factors, was associated with an increased risk of RE in patients not taking PPIs.

**Conclusions:**

RE, but not obesity, may have an independent association with the risk of ESBE in the Japanese population. Furthermore, obesity measures were not independent risks for RE. Interestingly, PPI-refractory RE and large WC were risk factors for ESBE ≥1 cm in patients taking PPIs.

## Background

The main risk for Barrett’s esophagus (BE) is considered to be gastroesophageal reflux disease (GERD) [1-4]. El-Serag reported that the incidence GERD has increased significantly in each of the last two decades in North America and Europe but not Asia [5]. However, the incidence of GERD is increasing in Japan as well as in the West [6]. These results suggest that the prevalence of GERD-associated BE will increase in Japan over the next few decades.

There is strong evidence from epidemiological studies conducted among Western populations that total obesity, as measured by body mass index (BMI), is associated with GERD symptoms, reflux esophagitis (RE) and esophageal adenocarcinoma [7,8]. Furthermore, these studies have shown that waist circumference (WC) and the waist-to-hip ratio (WHR), both of which measure abdominal fat, are risk factors for BE, independent of BMI [9,10].

In contrast, few studies have investigated the association between obesity and BE in a Japanese population. RE studies conducted in Japan have found a positive association between high BMI and RE [11,12], and RE is thought to be associated with the development of BE in the Japanese population [13,14]. In their retrospective study, Akiyama et al. reported a positive association between visceral obesity and BE in Japanese patients with non-alcoholic fatty liver disease, with visceral obesity being defined using visceral adipose tissue (VAT) measurements on abdominal computed tomographic images, but the strength of the risk of BE was very weak (odds ratio [OR], 1.0074; 95% confidence interval [CI], 1.0001-1.0147, p = 0.0472) [15]. Previous reports from Japan on the association between obesity and RE or BE have been limited to retrospective studies [13,15]. Therefore, it remains controversial whether obesity is actually an independent risk factor for BE in Japanese subjects.

In most of the BE cases identified in Japan, the lesions were very short, i.e., < 1 cm in length. Such cases are classified as ultra-short BE, although the diagnosis of this disease entity is difficult and highly unreliable [16-18]. One possible reason for the difficulty may be the use of different definitions of the gastroesophageal junction (GEJ), i.e., the distal end of the esophageal palisade vessels versus the proximal margin of the gastric folds [16,19,20]. As defined in the West, BE diagnosis requires histological confirmation of the specialized intestinal metaplasia (SIM). Thus, cases without histological confirmation of SIM are defined as endoscopically suspected BE (ESBE) in the US but are not regarded as BE [21]. Furthermore, the previous studies have included patients who were taking proton pump inhibitors (PPIs), which may induce the regression and normalization of BE [14,22,23]. Thus, the associations between obesity and BE should be investigated separately in patients taking and those not taking PPIs.

A recent study by Kramer et al. found no associations between BMI, WHR and short-segment BE, but a strong and statistically significant association between WHR and long-segment BE in the US [24]. In the current study, we focused on short-segment BE, and excluded ultra-short BE, and analyzed the association between this disease entity and obesity separately in patients taking and those not taking PPIs in Japan.

## Methods

### Patients

A hospital/clinic-based, cross-sectional, pilot study assessing the association between obesity and the development of BE was performed. Between November 2011 and June 2012, 1581 consecutive patients who underwent upper endoscopy at the Gastroenterology Division of Hyogo College of Medicine Hospital (n = 1114), one affiliated hospital (n = 327), and one clinic (n = 140) were enrolled in this study. Most patients were outpatients, and patients who had previously undergone upper gastrointestinal tract surgery, who had previously undergone endoscopy during this period, and those who were followed in the surveillance registry of BE in our department were excluded. At the affiliated hospital and the clinic, transnasal endoscopy with an ultrathin endoscope was performed only for medical check-up, while at our college hospital endoscopy was performed for various reasons, such as GERD symptoms (n = 200, 18.0%), detailed examination of gastric cancer or neoplasms (n = 78, 7.0%), follow-up study after endoscopic treatment for dysplasia and/or cancer (n = 36, 3.2%), and other reasons, including screening, follow-up for peptic ulcers, and so on. A standardized questionnaire was used to obtain a history from each subject regarding GERD symptoms, smoking and alcohol habits prior to endoscopy. Whether or not subjects were taking PPIs was determined by self-report and a review of the medical prescriptions in our database. The Ethics Committee of Hyogo College of Medicine and the affiliated hospital and clinic approved this study. Written, informed consent was obtained from all patients prior to this study.

### Endoscopy protocol

In Japan, the GEJ is defined as the distal limit of the lower esophageal palisade vessels [21], but in the West the GEJ is defined as the proximal margin or upper end of the gastric folds (Prague C&M criteria) [16]. In the present study, ESBE was diagnosed endoscopically as columnar-lined epithelium between the lower end of the palisade vessels of the lower esophagus and the squamocolumnar junction. However, if the palisade vessels could not be visualized clearly, the upper end of the gastric folds was regarded as the GEJ. When the ESBE length was considered to be 1 cm or more, the length was measured in comparison to the length of the endoscope. In contrast, when the length was considered to be < 1.0 cm, the ESBE length was judged in comparison to the biopsy forceps (the open forceps were about 7 mm in diameter; Radial Jaw™ 4: Boston Scientific Corporation, Natick, MA). If possible, at least one biopsy specimen was obtained from ESBE, > 5 mm in extent, in order to confirm the presence of SIM on histology. Atrophic gastritis was classified into 2 types according to the Kimura and Takemoto classification [25]: the closed type, as mild atrophic gastritis (C 1–3), and the open type, as severe atrophic gastritis (O 1–3). RE was diagnosed based on the Los Angeles (LA) classification [26], and individuals with LA classification grade A or more were considered positive for RE. These endoscopic data were prospectively collected.

### Measures of adiposity

Prior to endoscopy, we performed the following body measurements of the subjects. BMI was calculated as weight divided by the square of height (kg/m^2^). “Obesity” was defined as BMI ≥ 25.0 kg/m^2^, based on the criteria of the Japanese Society of Obesity [27]. A large WC based on the Japanese criteria (≥ 85 cm for men, ≥ 90 cm for women) was defined as an abnormal WC [28]. The high-risk category for WHR was defined as ≥ 0.9 for males and ≥ 0.85 for females, according to the definition of Ilanne-Parikka et al. [29].

### Symptoms and habits

GERD symptoms were defined as the presence of either heartburn or acid regurgitation at least once weekly. Also, the presence of a current alcohol (active consumption of any amount of alcohol) or cigarette (active smoking of any number of cigarettes) habit was determined by interview prior to the study. A PPI user was regarded as a patient who had continuously taken a PPI such as omeprazole (20 mg/day), lansoprazole (15 or 30 mg/day), rabeprazole (10 or 20 mg/day), or esomeprazole (20 mg/day) once a day beginning at least 2 months before this study.

### Statistical analysis

The data were assessed by the Mann–Whitney *U*-test for patients’ age and adiposity measures, and the chi-square test or Fisher’s exact test for the other variables between the two groups. Statistical significance was defined as a *p* value **<** 0.05. Odds ratios (ORs) and the corresponding 95% confidence intervals (CIs) for risk factors for ESBE and RE were calculated using StatView Version 5.0 for Macintosh (SAS Institute Inc., Cary, NC). We included all factors with p < 0.10 in unadjusted models in the multiple logistic regression model. Those factors with p > 0.05 in backwards stepwise regression were dropped from the final model.

## Results

### Patient characteristics

A total of 1581 individuals (921 men, 660 women) were enrolled in this study. Their mean age was 58.9 ± 14.7 years (range: 18 to 92 years). Of these, 1214 patients (76.8%) were not taking PPIs and, of these, 840 (69.2%) showed no evidence of ESBE on endoscopy and thus constituted our control group. On the other hand, the remaining 367 patients were regular PPI users, of whom 256 (69.8%) did not show ESBE and the remaining 111 (30.2%) had ESBE.

Among patients who were not taking PPIs (n = 1214), the prevalence of ESBE was 30.8% (374 cases): 25.2% (n = 306) for ultra-short ESBE, 5.4% (n = 65) for ESBE of ≥1 cm but <3 cm, and only 0.2% (n = 3) for long-segment ESBE (LSBE, ESBE ≥ 3 cm). On the other hand, that of ESBE among patients taking PPIs (367 cases) was 30.3%: 24.0% (n = 88) for ultra-short-segment ESBE, 5.2% (n = 19) for ESBE of ≥1 cm but <3 cm, and 1.1% (n = 4) for long-segment ESBE.

### Factors predicting development of ESBE ≥ 1 cm and RE

Because endoscopic diagnosis of ultra-short ESBE is difficult and highly unreliable, as mentioned above, patients with ultra-short ESBE were excluded from this evaluation.

As shown in Table 1, SIM was identified in patients with ESBE ≥ 1 cm (38.8%) among PPI non-users in whom a biopsy could be obtained, but did not differ from the frequency of SIM (44.4%) among regular PPI users who underwent biopsy. In patients not taking PPIs, there was no significant difference in age between the controls and the ESBE group. The percentage of males was significantly higher among patients with ESBE ≥ 1 cm than among controls (p = 0.03). RE was detected with significantly greater frequency in patients with ESBE ≥ 1 cm than among controls (p < 0.0001), both for patients taking and those not taking PPIs.

**Table 1:** Clinical characteristics of the patients with endoscopically suspected Barrett’s esophagus ≥ 1 cm

		**PPI non-users**			**PPI users**		
		**Controls**	**ESBE ≥ 1 cm**	***P***	**Controls**	**ESBE ≥ 1 cm**	***P***
Number		840 (69.2)	68 (5.6)	-	256 (69.8)	23 (6.3)	-
	SIM	-	19/49 (38.8)	-	-	8/18 (44.4)	-
Patients profile							
	Age, y	56.2 ± 14.8	58.3 ± 13.6	0.31	63.5 ± 15.3	67.5 ± 12.4	0.26
	Male sex	464 (55.2)	47 (69.1)	0.03	129 (50.4)	14 (60.9)	0.34
	Alcohol drinking	244 (29.0)	20 (29.4)	0.95	54 (21.1)	8 (34.8)	0.13
	Current smoking	173 (20.6)	14 (20.6)	0.99	34 (13.3)	3 (13.0)	> 0.99
	GERD symptoms	126 (15.0)	12 (17.6)	0.56	74 (28.9)	9 (39.1)	0.30
Endoscopic findings							
	Reflux esophagitis	68 (8.1)	17 (25.0)	<0.0001	22 (8.6)	8 (34.8)	0.0001
	Gastric atrophy (Open-type)	158 (18.8)	16 (23.5)	0.34	96 (37.5)	6 (26.1)	0.28

*PPI* proton pump inhibitor, *ESBE* endoscopically suspected Barrett’s esophagus, *SIM* specialized intestinal metaplasia, *GERD* gastroesophageal reflux disease, *NS* not significant.

Among both patients taking and those not taking PPIs, all adiposity measures including BMI, WC, and WHR in patients with ESBE ≥ 1 cm were not significantly different from those among controls (Table 2). However, the prevalence of large WC was significantly higher in patients with BE ≥1 cm than in the controls (p < 0.0001) among patients regularly taking PPIs, but not among those not taking PPIs.

**Table 2:** Distributions of BMI, WC and WHR among cases and controls

**A. Patients not taking PPIs**					
**Adiposity measures**	**Controls**	**(%)**	**ESBE ≥ 1 cm**	**(%)**	***P *value**
BMI, kg/m^2^	21.8 ± 3.5	(17.2)^*^	22.9 ± 2.64	(30.4)^*^	0.11
WC, cm	80.3 ± 10.8	(23.4)^**^	82.7 ± 8.68	(60.9)^**^	<0.0001
WHR	0.87 ± 0.08	(49.6)^†^	0.90 ± 0.04	(42.9)^†^	0.55
**B. Patients regularly taking PPIs**					
**Adiposity measures**	**Controls**	**(%)**	**ESBE ≥ 1 cm**	**(%)**	***P *value**
BMI, kg/m^2^	22.4 ± 3.5	(19.5)^*^	22.3 ± 3.3	(19.1)^*^	0.94
WC, cm	80.8 ± 10.2	(29.8)^**^	81.5 ± 10.1	(36.8)^**^	0.27
WHR	0.88 ± 0.08	(46.7)^†^	0.88 ± 0.06	(42.3)^†^	0.54

*BMI* body mass index, *WC* waist circumference, *WHR* waist-to-hip ratio, *PPI* proton pump inhibitor.

ESBE endoscopically suspected Barrett’s esophagus.

*P* values indicate the comparison in the prevalence between controls and ESBE ≥ 1 cm.

* The prevalence indicates body mass index ≥ 25 kg/m^2^.

** The prevalence indicates a large waist circumference defined as ≥ 85 cm for males and ≥ 90 cm for females.

† The prevalence indicates a large waist-to-hip ratio defined as ≥ 0.9 for males and ≥ 0.85 for females.

When analyzing the strength of the association between obesity and ESBE development on multivariate logistic regression analysis, RE was an independent risk factor for ESBE ≥ 1 cm in both patients who were not taking PPIs (OR = 3.48, 95% CI = 1.89-6.41, p < 0.0001) and those who were regularly taking PPIs (OR = 5.67, 95% CI = 2.17-14.86, p = 0.0004) (Table 3). Intriguingly, a large WC was a significant risk factor for BE ≥ 1 cm (OR = 5.09, 95%CI = 2.04-12.72, p = 0.0005) only among regular PPI users.

**Table 3:** Predictors of the development of endoscopically suspected Barrett’s esophagus

**A. Patients not taking proton pump inhibitors**						
	**Univariate**			**Multivariate**		
	**OR**	**95% CI**	***P *value**	**OR**	**95% CI**	***P *value**
Male sex	1.81	1.07–3.09	0.03	1.61	0.94–2.77	0.08
Reflux esophagitis	3.78	2.07–6.91	<0.0001	3.48	1.89–6.41	<0.0001
**B. Patients regularly taking proton pump inhibitors**						
	**Univariate**			**Multivariate**		
	**OR**	**95% CI**	***P *value**	**OR**	**95% CI**	***P *value**
Reflux esophagitis	5.67	2.17–14.86	0.0004	5.67	2.17–14.86	0.0004
Large waist circumference *	5.08	2.10–12.33	0.0003	5.09	2.04–12.72	0.0005

*OR* odds ratio, *CI* confidence interval.

* Large waist circumference is defined as ≥ 85 cm for males and ≥ 90 cm for females.

Next, the relationship between RE and the patient characteristics was investigated in patients not taking PPIs. Male sex (p < 0.0001) and alcohol drinking (p = 0.049) were significantly associated with the development of RE (data not shown), whereas gastric atrophy significantly reduced the development (p = 0.02). In keeping with the findings from previous studies in the West [7,8] and Japan [11,12] in which RE was significantly associated with obesity, all adiposity measures in the present study, i.e., BMI, WC, and WHR, were significantly greater in patients with RE than in those without (p < 0.0001, p < 0.0001, and p = 0.0006, respectively) (Table 4). Moreover, all obesity indices were associated with RE (p = 0.001, p = 0.0003, and p = 0.02, respectively). In the multivariate analyses, however, only male sex, but none of the obesity measures, was a risk factor for RE (Table 5). Among patients taking PPIs, only GERD symptoms were significantly associated with RE development (p = 0.004, data not shown).

**Table 4:** Relationship between reflux esophagitis and obesity in patients not taking PPIs

	**Reflux esophagitis**		
**Adiposity measures**	**Positive**	**Negative**	***P *value**
BMI, kg/m^2^	23.6 ± 3.6	22.4 ± 3.4	<0.0001
WC, cm	84.5 ± 10.0	80.8 ± 10.1	<0.0001
WHR	0.90 ± 0.07	0.87 ± 0.08	0.0006

*PPI* proton pump inhibitor, *BMI* body mass index, *WC* waist circumference, *WHR* waist-to-hip ratio.

**Table 5:** Predictors for the development of reflux esophagitis in patients who are not taking PPIs

	**Univariate**			**Multivariate**		
	**OR**	**95% CI**	***P *value**	**OR**	**95% CI**	***P *value**
Male sex	2.57	1.68–3.93	< 0.0001	2.22	1.25–3.92	0.006
Alcohol drinking	1.47	1.01–2.15	0.05	1.10	0.68–1.80	0.70
Gastric atrophy (Open-type)	0.54	0.31–0.93	0.03	0.57	0.32–1.03	0.06
BMI (25≤)	1.95	1.31–2.92	0.001	1.51	0.85–2.70	0.16
WC *	1.98	1.37–2.87	0.0003	1.04	0.56–1.94	0.89
WHR **	1.70	1.09–2.66	0.02	1.57	0.92–2.67	0.10

*PPI* proton pump inhibitor, *OR* odds ratio, *CI* confidence interval, *BMI* body mass index, *WC* waist circumference, *WHR* waist-to-hip ratio.

* Large waist circumference is defined as 85 cm ≤ for males and 90 cm ≤ for females.

** Large waist-hip ratio is defined as ≥ 0.9 for males and ≥ 0.85 for females.

## Discussion

A recent study in the US reported that high WHR, but not BMI, was associated with a significant increase in the risk of LSBE but not short-segment BE in white men [24]. The present study is the first cross-sectional study using prospective endoscopic data collection to evaluate the association between obesity and the risk of ESBE, especially short-segment ESBE, in a Japanese population. The pathognomonic point of this study is that the risk factors for ESBE ≥ 1 cm, excluding ultra-short ESBE for which endoscopic diagnosis is highly unreliable, were investigated separately in subjects taking and those not taking PPIs in order to exclude the possibility of a drug affecting the pathophysiology of BE.

The natural history of GERD, including RE, is incompletely understood because very few well-designed prospective studies and endoscopic studies in general populations have been performed [30]. In the current study, RE was a significant risk factor for ESBE development. In two retrospective studies from Japan, RE was shown to be associated with ESBE [13,14] and also with the progression of ESBE using a multivariate model [13]. Therefore, the present findings support these previous results from Japan. However, these studies unfortunately included ultra-short ESBE. According to the previous reports [14,22,23], PPI administration might heal RE and induce short BE to regress. Of 367 patients who were regularly taking PPIs, 33 subjects (9.0%) showed RE in the present study (data not shown). It has been reported that the proportion of PPI-refractory RE patients is approximately 7–15% [31], a finding that is consistent with the present result. Furthermore, the incidence of PPI treatment-resistant RE was significantly higher in patients with ESBE ≥ 1 cm (34.8%) than in those with ultra-short ESBE (3.4%, 3 of 88) (p = 0.0001, data not shown). These results indicate that PPI-refractory RE may be strongly associated with an increasing risk of developing ESBE ≥ 1 cm.

In the current study, all adiposity measures were significantly associated with RE, whereas obesity was not a risk factor for increased BE. Observational studies from Asia have reported a consistent association between WC or VAT and RE [32,33]. Two studies of RE in a Japanese population showed that abdominal obesity may be an important risk factor for RE in males, but not in females [34,35], but another report did not show this association [36], which has led to some controversy about the association between obesity and RE. The present study clearly demonstrated that RE was an independent risk factor for ESBE, while obesity was not a risk factor for RE. Only male sex was an independent risk factor for RE development. As reported previously from Western countries, differences in body fat distribution, rather than simple obesity as measured by the BMI, may cause GERD or BE, with abdominal fat deposition leading to an increase in intra-abdominal pressure and GERD. Corley et al. also noted that WC may predict the risk of BE or esophageal adenocarcinoma better than BMI [9]. It is also true that there are differences in the demographic distribution of BE by race and ethnicity [37]. The difference in the association between obesity and RE or BE might be explained, at least in part, by ethnic differences in the obesity pattern, especially the pattern of visceral adipose tissue deposition [15]. Intriguingly, in patients who are taking PPIs, large WC was a risk factor for ESBE ≥ 1 cm but not for ultra-short ESBE (data not shown), although the reason for this finding is unclear. The differences in risk factors between ultra-short and BE ≥ 1 cm indicate that the pathophysiologies of these two conditions may be different [38]. One possible mechanism for the development of ESBE ≥ 1 cm in Japanese subjects may be that visceral obesity as measured by WC is associated with severe esophageal acid exposure that cannot be sufficiently inhibited by PPI treatment alone. Therefore, 24-hour esophageal pH monitoring will be needed to clarify the mechanism in such patients.

Male sex was not a significant risk factor for ESBE with or without PPIs (OR 1.61, 95% CI = 0.94-2.77, p = 0.08) in this study, although it was associated with an increased risk of RE development. Male sex has been reported to be an important predictive factor for BE [10,37]. Generally, the appropriate WC in females differs from that in males because there is a difference in the preferential accumulation of VAT between females and males [39,40]. Thus, the risk factors for BE development may be different between females and males. However, no association between obesity and RE or ESBE was found in either females or males in the present study (data not shown). Further investigation may be required using a larger series of samples to determine the association between gender and BE.

There was no significant difference in the prevalence of ESBE, including ultra-short ESBE, between patients taking PPIs (30.2%, 111 of 367) and those not taking PPIs (30.8%, 374 of 1214). The prevalence rates of endoscopically suspected LSBE were only 1.1% and 0.2% in patients taking PPIs and those not taking PPIs, respectively; therefore, the remaining cases were mostly short-segment ESBE, especially ultra-short ESBE < 1 cm. These findings are consistent with reports from Japan and Asia, in which SSBE was shown to be more common and LSBE more rare than in Western countries [41,42]. It remains unclear why the prevalence of LSBE is different between Japan and the West. Some possible reasons for this difference include differences in genetic susceptibility, genetic polymorphisms, diet or other clinical/nutritional factors, and environmental influences (socio-economic status) [43], along with differences in the definition of the BE and GEJ.

The frequency of SIM increased with longer BE, a finding that is consistent with recent reports [44,45]. In our study, biopsy sampling was not done based on the Seattle biopsy protocol, which is a widely accepted method for performing surveillance biopsies [46], and this omission presumably increased the chance of bias inclusion. However, SIM was detected in 38.8% (19 of 49) of patients with ESBE ≥ 1 cm who were not taking PPIs, even in those with ultra-short BE (20.2%, 23 of 114), and this prevalence was higher compared to the finding of Westerhoff et al. [44]. It may be problematic to distinguish metaplasia of the gastric cardia from metaplasia of the BE and GEJ [10]. We previously reported that SIM obtained from SSBE, mostly ultra-short BE, was distinct from metaplasia in the cardia and the antrum from a practical molecular pathology standpoint [47]. More recently, it was reported that palisade vessels are a specific, original, and useful histologic marker of tissue originating from the esophagus [48], thus supporting the Japanese criteria in which the distal end of the esophageal palisade vessels is considered the GEJ. Taking the findings of these reports into consideration, we believe that ESBE with palisade vessels as investigated in the present study should actually be diagnosed as BE irrespective of the presence or absence of SIM based on the Japanese [49] or British criteria [50].

The present study had several potential limitations. A possible weakness is that only ESBE without histological confirmation of SIM was evaluated, in contrast with the American criteria [21]. Although we examined the effect of obesity on the risk of BE with SIM, there was no significant association between them because of the limited number of cases of BE ≥ 1 cm with SIM (n = 19). Further study will be needed to accurately clarify this association in a Japanese population as well as in the West. Second, this study was a hospital/clinic-based study, so the pooled data might not represent the general population. In addition, the sample size was relatively small. Third, it was not established whether any of the endoscopic BE patients had a hiatus hernia, which is thought to be a risk factor of BE development, as shown previously [14,15,20]. Fourth, *H. pylori* infection, which appears to have an inverse association with BE, was not evaluated [40,51]. However, the present study showed that gastric atrophy, which is caused by *H. pylori* infection, was not related to BE development, a finding that is consistent with the previous study [13]. Additionally, we did not analyze the administration of nonsteroidal anti-inflammatory drugs, including aspirin, which have been significantly associated with a risk of BE [52].

## Conclusions

In conclusion, simple and visceral obesity were not risk factors for ESBE in the present study. In fact, RE was the only one of the parameters studied that was a significant risk factor for ESBE independent of obesity. Interestingly, PPI-refractory RE and large WC in regular PPI users were strongly associated with increasing risk of ESBE ≥ 1 cm. Further studies with a larger sample size will be needed to clarify the reason for this lack of association between obesity and ESBE in the Japanese population.

## Abbreviations

BE: Barrett’s esophagus; ESBE: Endoscopically suspected BE; BMI: Body mass index; WC: Waist circumference; WHR: Waist-to-hip ratio; VAT: Visceral adipose tissue; PPI: Proton pump inhibitor; RE: Reflux esophagitis; GERD: Gastroesophageal reflux disease; GEJ: Gastroesophageal junction; SIM: Specialized intestinal metaplasia; OR: Odds ratio; CI: Confidence interval.

## Competing interests

The authors declare that they have no competing interests.

## Authors’ contributions

JW provided major input into the conceptual development of the study, analyzed the dataset, wrote the manuscript and supervised all investigations. All authors performed esophagogastroduodenoscopy according to our endoscopy protocol and measured adiposity measures in the patients. NK input all the data to a database. JW and HM had full access to all of the data (including statistical reports and tables) in the study and take responsibility for the integrity of the data and the accuracy of the data analysis. All authors read and approved the final manuscript.

## Pre-publication history

The pre-publication history for this paper can be accessed here:

http://www.biomedcentral.com/1471-230X/13/143/prepub
